# Evaluating the Impact of Cultivar and Processing on Pulse Off‐Flavor Through Descriptive Analysis, GC–MS, and E‐Nose

**DOI:** 10.1111/1750-3841.70610

**Published:** 2025-10-24

**Authors:** Kaveri Ponskhe, Aubrey DuBois, Lili Towa, Sharon Hooper, Karen Cichy, Emily J. Mayhew

**Affiliations:** ^1^ Department of Food Science and Human Nutrition Michigan State University East Lansing Michigan USA; ^2^ Alpha MOS Toulouse France; ^3^ Department of Plant, Soil and Microbial Sciences Michigan State University East Lansing Michigan USA; ^4^ Sugarbeet and Bean Research Unit USDA‐ARS East Lansing Michigan USA

## Abstract

Pulses are nutrient‐dense and have a low carbon and water footprint but remain underutilized in the United States. A potential strategy to boost pulse consumption involves milling pulses into flour and incorporating them into convenience products traditionally made from wheat flour. However, acceptance of pulse‐based products often decreases due to beany off‐flavors. Hence, addressing off‐flavors—commonly described as green/vegetative, musty/earthy, or beany—is essential for sustained adoption. This study evaluated the impact of cultivar selection and processing methods (boiling, roasting) to reduce off‐flavors in eight pulse cultivars using descriptive analysis (DA) and rapid volatile profiling with gas chromatography–mass spectrometry (GC–MS) and electronic nose (e‐nose). DA revealed significant differences (*p* < 0.05) across cultivars and processing treatments for 20 sensory attributes, with roasting reducing green/vegetative and earthy/mushroom/musty off‐flavors but increasing beany characteristics, especially in roasted navy bean flour. We identified eight key volatiles via GC–MS, including ketones, aldehydes, and alcohols, which were strongly correlated with green/vegetative and musty/earthy flavors. However, GC–MS had limitations in predicting beany off‐flavors, likely due to the chosen targeted analytical approach. In contrast, the untargeted e‐nose approach effectively identified discriminant ions that correlated with sensory attributes such as toasted and beany odors in roasted flours. E‐nose data aligned better with DA results, highlighting its potential as a first screening tool for rapid flavor profiling. Findings highlight the importance of refining pretreatment methods and selecting cultivars with milder flavors. E‐nose and GC–MS can be used to optimize the sensory quality of pulse flour, supporting increased consumer acceptance of pulse‐based products.

**Practical Applications:**

Despite widespread production of common beans, their consumption is often limited due to off‐flavors. While pulse flavor research has largely focused on peas, chickpeas, and faba beans, there is a need to explore cultivar selection and processing strategies for beans. This study demonstrates that lighter‐colored bean cultivars and pretreatment with roasting can reduce known off‐flavors. Additionally, e‐nose offers a rapid alternative compared to traditional sensory and GC–MS methods, helping breeders and product developers to efficiently screen large sample sets for off‐flavors. These findings can support the development of pulse‐based products with reduced off‐flavors, ultimately improving consumer acceptance and consumption.

## 1 Introduction

Pulses are edible seeds harvested dried from legume plants (Fabaceae). Common pulse types include *Phaseolus vulgaris* (dry beans such as kidney, navy, and pinto beans), *Cicer arietinum* (chickpeas), and *Pisum sativum* (peas) (FAO 1994). Incorporating pulses into the daily diet has been associated with numerous health benefits, including reduced risks of heart disease, type 2 diabetes, and colon cancer, due to their high protein, fiber, and folate content (Geil and Anderson 1994; Michels et al. 2006). Environmentally, pulses improve soil health through nitrogen fixation (Reckling et al. 2016) and have a significantly lower carbon footprint than animal‐based proteins (Clune et al. 2017).

Despite these benefits, pulse consumption in the United States remains low, with 83% of Americans consuming pulses below the recommended intake of 2.5 cups/week according to the Dietary Guidelines for Americans (Dietary Guidelines Advisory Committee 2004; Garden‐Robinson and West 2023; Mitchell et al. 2021; Sadohara et al. 2022). Barriers include long cooking times, limited preparation knowledge, and aversion to the taste and texture of pulses (Doma et al. 2019; Winham et al. 2020).

A potentially effective strategy to address the consumer barrier related to long cooking times is to incorporate pulse flours into products traditionally made with wheat flour, such as pasta or crackers. However, sensory challenges—especially off‐flavors—limit their sustained adoption (Sadohara et al. 2022). These off‐flavors, often described as “beany,” include sub‐character notes such as musty, earthy, and green aromas (Chigwedere et al. 2022; Troszyńska et al. 2011; Vara‐Ubol et al. 2004). In this study, these sub‐character notes are referred to as “known off‐flavors.” While future consumer studies are needed to assess their impact on acceptance, this study does not classify beany notes as off‐flavors, as their acceptability may vary depending on product context (Chigwedere et al. 2022).

Volatile organic compounds (VOCs) responsible for off‐flavors, including aldehydes, alcohols, ketones, nitrogenous, and sulfur‐containing compounds, can be minimized through cultivar selection and processing optimization (Roland et al. 2017). Hence, combining instrumental analysis with sensory evaluation is ideal for generating a comprehensive understanding of off‐flavors in pulses (Viana and English 2021). However, the time and cost investment for sensory panel training limits its use for large sample sets (Shurmer and Gardner 1992). To overcome these limitations, instrumental methods such as headspace solid‐phase microextraction gas chromatography–mass spectrometry (HS‐SPME–GC–MS) are widely used due to their high sensitivity and ability to identify and quantify individual VOCs in pulses (Karolkowski et al. 2021; Khrisanapant et al. 2019). The electronic nose (e‐nose) has emerged as a promising alternative that rapidly generates an odor fingerprint reflective of overall aroma, using either flash GC or sensor arrays (Wilson and Baietto 2009). Given the time‐consuming nature of sensory testing, comparative studies evaluating the ability of traditional and novel instrumental techniques to predict sensory profiles can streamline the process of rapid sample screening.

Therefore, this study aims to (1) characterize the impact of cultivar and processing on sensory attributes of pulses through sensory descriptive analysis and (2) identify chemical markers associated with off‐flavors using instrumental techniques (gas chromatography–mass spectrometry [GC–MS] and e‐nose). By examining the effects of cultivar variation and processing methods (boiling and roasting), the study seeks to identify cultivars with milder flavor profiles and evaluate the sensory trade‐offs involved in processing to reduce off‐flavors. These findings aim to enhance the sensory quality and consumer acceptance of pulse‐based food products.

## 2 Materials and Methods

### 2.1 Germplasm Selection and Seed Production

**FIGURE 1:**
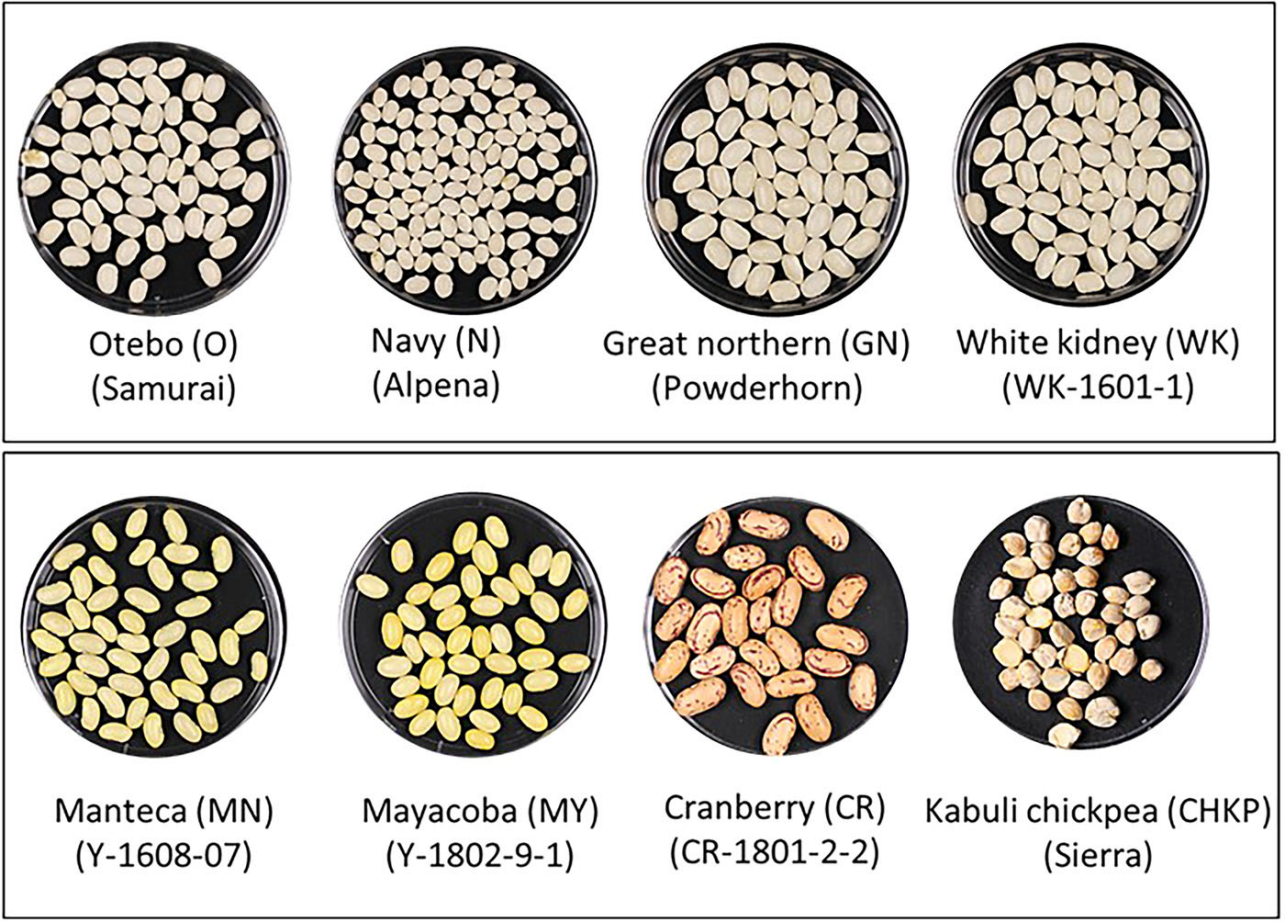
Image of the eight cultivars included in this study, arranged by market class, abbreviation, and corresponding genotypes (shown in parentheses).

The dry bean market classes selected for this study with their respective abbreviations and cultivar (cv.) or genotypes are listed as follows: Navy (N, cv. ‘Alpena’); Otebo (O, cv. ‘Samurai’); Great Northern (GN, cv. ‘Powderhorn’); White Kidney (WK, cv. ‘WK 1601‐1’); Mayacoba (MY, cv. ‘Y 1802‐9‐1’); Manteca (MN, cv. ‘Y1608‐07’); and Cranberry (CR, cv. ‘CR1801‐2‐2’) (Figure 1). The rationale for the selection of these beans was based on their adaptation to Michigan's agricultural conditions, seed yield potential, and representation across market classes. For the potential higher acceptance of pulse flour, cultivars with white or lighter seed coat colors, such as Navy, Otebo, Great Northern, and White Kidney, were chosen.

**FIGURE 2:**
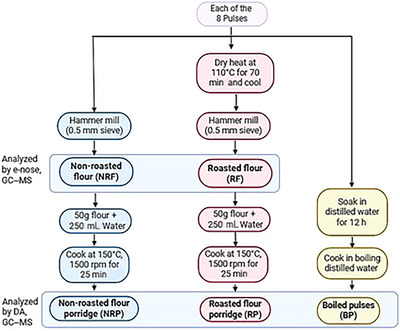
Flowchart of preparation methods for five types of samples. Electronic nose (e‐nose) and gas chromatography–mass spectrometry (GC–MS) analyses were conducted on NRF and RF samples; GC–MS and descriptive analysis (DA) were performed on NRP, RP, and BP samples from each of the eight pulse types of Navy, Otebo, Great Northern, White Kidney, Mayacoba, Manteca, Cranberry, and Chickpea.

These beans were cultivated at the Michigan State University Montcalm Research Center in Entrican, Michigan, during the year 2022. The seeds were sown in a randomized complete block design with three field replicates, with plots consisting of four 6.1‐m rows, where the center rows contained the experimental lines and the outer rows were standard bordered with kidney beans. Field maintenance practices included weed control, fertilization, and insect management, with supplemental irrigation as needed. The seeds were harvested on September 29 using a Hege 140 plot combine harvester. Postharvest, the seeds were cleaned manually to remove debris and stored in paper bags at room temperature for further analysis. Additionally, a Kabuli Chickpea (CHKP, cv. ‘Sierra’) obtained commercially, grown in 2022 on a Montana commercial farm, was chosen in this study for its industrial significance in US production. Non‐roasted pulse flour (NRF), non‐roasted pulse flour porridge (NRP), roasted pulse flour (RF), roasted pulse flour porridge (RP), and boiled pulses (BPs) were produced from each of the eight pulse genotypes (Figure 2).

#### 2.1.1 Pulse Flour Production

The pulses were rinsed under distilled water, spread on a tray lined with paper towels, and allowed to air dry for 12 h. Some of the cleaned and dried pulses were roasted by dry heat in an oven (Isotemp Gravity Oven, 100 L; Fisher Scientific, Waltham, MA, USA) at 110°C for 70 min, then allowed to cool for 4 h. All sample preparation steps were conducted under standard laboratory conditions, with a temperature of 22°C, relative humidity between 30% and 40%, and atmospheric pressure of 1 atm (101.3 kPa).

Once dried, the non‐roasted and roasted seeds from each of the eight pulse varieties were milled into flour using a hammer mill (Polymix Laboratory Grinding Mills, PX‐MFC 90 D, Kinematica; Bohemia, NY, USA), fitted with a 0.5‐mm sieve to produce NRF and RF samples.

#### 2.1.2 Pulse Porridge and BP Preparation

Both NRF and RF samples were used to prepare porridges for sensory and volatile analyses using the same procedure to understand the cooked properties of the pulse flour. To prepare the porridge, 50 g of pulse flour (NRF or RF) was mixed with 250 mL of water to form a slurry and stirred for 7 min. An additional 300 mL of distilled water was then added, and the mixture was cooked on a magnetic hotplate stirrer (PRO 4‐Channel LCD Digital Magnetic Stirrer; MSE Supplies LLC, Tucson, AZ, USA) with the surface temperature setting at 150°C and mixed at 1500 rpm for 25 min, producing NRP and RP samples. Based on pilot experiments, these conditions were optimized to ensure consistent mixing and cooking of NRF or RF, resulting in homogenous porridge samples suitable for sensory and volatile analysis. BP samples were prepared by soaking pulses in distilled water for 12 h at room temperature, followed by boiling on a portable induction cooktop (1800 W Portable Induction Cooktop; Duxtop, Durham, NC, USA) until fully cooked (Figure 2). Cooking times were determined using a Mattson pin drop cooker (Department of Physics and Astronomy Machine Shop, Michigan State University, East Lansing, MI, USA) as follows: Otebo, 16 min; Navy, 24 min; Great Northern, 23 min; White Kidney, 30 min; Chickpea, 45 min; Manteca, 20 min; Mayacoba, 33 min; and Cranberry, 50 min. NRP, RP, and BP samples were prepared fresh on the day of testing for sensory and GC–MS volatile analysis. NRF and RF samples were stored in sealed bags after milling under refrigeration at 2°C to reduce volatile loss (Akkad et al. 2022).

### 2.2 Descriptive Analysis (DA)

The NRP, RP, and BP samples were characterized for their sensory profile using quantitative DA (ISO 11035:1994). Since flour cannot be directly consumed by human panelists, to understand the characteristics of pulse flour in its simplest form, pulse porridges from the pulse flour and BPs from raw seeds were prepared as described above for sensory assessment.

Sensory panelists were recruited and then screened by a series of tests to evaluate each candidate's ability to identify basic tastes, rank taste intensities, recognize aromas, and generate descriptors for canned chickpeas and canned pinto bean samples to assess their taste acuity and verbal ability to participate in a 6‐week DA panel. Candidates who demonstrated consistent performance across these tests were selected as panelists for further training. The panel consisted of nine panelists (two males, seven females) aged 18–41. The panelists underwent a training program consisting of 27 one‐hour sessions. Sessions 1–3 focused on the introduction to DA methodology and sensory modalities (appearance, aroma, taste, flavor, and aftertaste). Sessions 4–6 involved group discussions to generate, define, and refine relevant sensory attributes and define the rinse protocol. The rinse procedure included the following steps: expectorating the sample, rinsing with room temperature water and expectorating the water, biting into a cracker to cleanse the palate and expectorating, and, finally, rinsing with room temperature water and expectorating the water again. Each day, a rotating, balanced subset of pulse samples from the experiment was provided for panel training and practice, including NRP, RP, and BP samples every session. In Sessions 7–9, references were generated based on their suitability to the sample matrix and then refined and selected for each attribute on the lexicon. In the next three sessions, these references were anchored using the sample set to define the scale range rather than a universal scale. The attributes generated and evaluated by the panel, along with references, definitions, and sample evaluation instructions, are listed in Table 1. Sessions 13–15 involved scaling practice (0–15 scale) as a group using anchored references beginning with taste to emphasize alignment of intensity perception across panelists. The next six sessions focused on calibration of appearance, aroma, flavor, and aftertaste, where panelists rated samples individually, followed by group discussions to identify outliers, discrepancies, and clarification of attribute interpretations. The last four sessions emphasized consistency and repeatability by using references consistently across sample types.

**TABLE 1:** Lexicon used to characterize pulse samples, including sensory attributes used in the descriptive analysis, corresponding codes, definitions, references, evaluation procedures for pulse samples, and reference ratings on a 0–15 scale. Attributes are organized by sensory modality.

Attribute	Abbreviation	Definition	Reference	Reference rating
Appearance Protocol for sample: Lid off and evaluate each sample cup over white paper and use the respective color swatches as references for assessment				
Value	color_value	The value of the sample from light to dark	Greyscale (Munsell Color Company)	1‐3‐4‐7‐9‐12‐14
Saturation	color_saturation	The saturation of the sample from dull to bright	10YR hue page (Munsell Color Company)	1‐3‐5‐7‐9‐11‐13‐14

Aroma Protocol for sample: Shake samples and crack the corner of the lid to sniff				
Kidney bean	aroma_kidney.bean	Aroma of canned kidney beans	Canned kidney beans	13
Chickpeas	aroma_chickpeas	Aroma of canned chickpeas	Canned chickpeas	14
Pinto beans	aroma_pinto.bean	Sweet aroma of canned pinto beans	Canned pinto beans	11
Great northern beans	aroma_great. northern.bean	Sour aroma of canned great northern beans	Canned great northern beans	11
Mushroom	aroma_mushroom	Musty aroma of fresh mushroom	Uncooked sliced mushroom	14
Boiled potato	aroma_boiled.potato	Aroma of peeled, boiled and mashed potato	Boiled potato	11.5
Boiled rice	aroma_boiled.rice	Aroma of boiled rice	Boiled rice	12
Toasted bread	aroma_ toasted.bread	Aroma of fresh white toasted bread	White toasted bread	12
Tofu	aroma_tofu	Fermented aroma of uncooked tofu	Firm tofu	10
Grainy	aroma_grainy	Aroma of cooked grains	Cream of wheat	10

Aroma‐by‐mouth/flavor Protocol for sample: Chew one piece of the whole beans thoroughly with back teeth for 3 s. Move a spoonful of porridge thoroughly around the mouth for 3 s.				
Kidney bean	flavor_kidney.bean	Flavor of canned kidney beans	Canned kidney beans	13
Chickpeas	flavor_chickpeas	Flavor of canned chickpeas	Canned chickpeas	13
Pinto beans	flavor_pinto.bean	Sweet flavor of canned pinto beans	Canned pinto beans	11
Great northern beans	flavor_great. northern.bean	Sour flavor of canned great northern beans	Canned great northern beans	12
Mushroom	flavor_mushroom	Musty flavor of fresh mushroom	Uncooked sliced mushroom	14.5
Boiled potato	flavor_boiled.potato	Aroma of peeled, boiled and mashed potato	Boiled potato	8.8
Tofu	flavor_tofu	Fermented flavor of uncooked tofu	Raw tofu	7
Vegetable	flavor_vegetable	Flavor of cooked green vegetables	Canned green bean	13

Taste Protocol for sample: Chew one piece of the whole beans thoroughly with back teeth for 3 s. Move a spoonful of porridge thoroughly around the mouth for 3 s.				
Sour	taste_sour	Sour taste of Citric acid solution	0.05% Citric acid Solution	11
Umami	taste_umami	Umami taste of MSG solution	0.05% MSG solution	11.2
Bitter	taste_bitter	Bitter taste of caffeine solution	0.05% Caffeine Solution	9

Aftertaste Protocol for sample: Chew one piece of the whole beans thoroughly with back teeth for 5 s and expectorate. Move a spoonful of porridge thoroughly around the mouth for 5 s and expectorate.				
Astringent	aftertaste_astringent	Lingering dryness after swallowing	0.05% Alum solution	13.5
Bitter	aftertaste_bitter	Bitter aftertaste of caffeine solution	0.05% Caffeine Solution	9

After their training, the panelists evaluated samples in individual sensory booths in duplicate using the RedJade sensory software (RedJade Sensory Solutions LLC, Pleasant Hill, CA, USA). The NRP, RP, and BP samples from each of the eight pulse cultivars were presented in 5‐ounce (oz) plastic cups with lids, following a randomized complete block design, blinded with random three‐digit codes, across four evaluation sessions on four consecutive days. Before each evaluation session, panelists were instructed to recalibrate themselves using freshly prepared reference samples. These references were labeled with their identity and served in 5‐oz plastic cups with lids. The panelists rated attribute intensities of the samples on a questionnaire using a continuous, visual analog scale from 0 to 15 anchored at the ends by none and strong for most attributes, except for saturation, which was anchored by dull and bright.

This study was approved by the Michigan State University Institutional Review Board (IRB #STUDY00008203), and informed consent was obtained from all panelists prior to participation.

### 2.3 HS‐SPME–GC–MS Analysis

NRF, NRP, RF, RP, and BP samples were analyzed for HS‐SPME–GC–MS. Volatile profiles of all five samples were obtained using the same equipment, procedure, and conditions.

The following quantities of each sample were placed in 20‐mL headspace vials: 2 g each of NRF and RF, 5 g of mashed BP, and 5 g of NRP and RP (each porridge mixed with 1 g NaCl). NaCl addition enhanced volatile extraction by lowering the partitioning coefficient (K) for some analytes and increasing their concentration in the headspace (Westland 2021).

Samples were then analyzed by the HS‐SPME–GC–MS method described previously (Ponkshe et al. 2025). Briefly, samples were first equilibrated at 50°C for 30 min, followed by exposing a carboxen/polydimethylsiloxane/divinylbenzene (CAR/PDMS/DVB) 2 cm, 30/50 µm (Supelco; Sigma–Aldrich, St. Louis, MO, USA) SPME fiber to the headspace for an additional 30 min at 50°C. Volatile compounds were desorbed for 20 s in a split/splitless injector port (200°C) of a gas chromatograph (Agilent 6890 Gas Chromatograph; Hewlett‐Packard Co., Wilmington, DE, USA) and separated on a 30 m × 0.25 mm i.d. HP‐5 (Hewlett‐Packard) capillary column (0.25 µm) with helium carrier gas at a ramped flow rate, initially 1.2 mL/min and then increased by 1 mL/min to a final flow rate of 1.8 mL/min. The initial GC oven temperature was set at 32°C and increased to 60°C at a rate of 20°C/min. It was then ramped to 150°C at a rate of 50°C/min, followed by a final increase to 280°C at a rate of 70°C/min, where it was held for 2 min. The total run time for the analysis was 7.4 min. Detection was carried out using TOF‐MS (LECO Pegasus III) with electron ionization at 70 eV and a mass range of 29–400 *m*/*z*. Volatile compounds were identified through comparisons with the National Institute of Standards and Technology (NIST) mass spectra library database (V.05) and/or by matching retention times of authenticated standards. The following volatiles were identified using authenticated pure commercial standards: 2‐butanone, 2‐methyl butanal, butanol, 2‐ethylfuran, 3‐methylbutanol, dimethyl disulfide, 1‐pentanol, hexanal, (*E*)‐2‐hexenal, 1‐hexanol, o‐xylene, 2‐heptanone, styrene, heptanal, methional, 2,5‐dimethyl pyrazine, benzaldehyde, 1‐octen‐3‐ol, 6‐methyl‐5‐hepten‐2‐one, octanal, decane, l‐limonene, nonanal, decanal, and geosmin, all obtained from Sigma–Aldrich.

The peak areas of volatiles were collected from the HS‐SPME–GC–MS analysis of each sample and calculated as the average of triplicate area‐under‐the‐curve (AUC) measurements and reported for a single *m*/*z* (mass‐to‐charge ratio) corresponding to the unique mass (Ponkshe et al. 2025) (Park et al. 2024). A blank run was performed after each sample.

### 2.4 E‐Nose Analysis

The volatile profile analysis of pulse flours was also conducted using an ultrafast chromatographic system, Heracles Neo (Alpha MOS, Toulouse, France). The instrument was equipped with two metal capillary columns working in parallel mode and characterized by different polarity and stationary phase: a nonpolar column (MXT5: 5% diphenyl, 95% methylpolysiloxane, 10 m length, and 180 µm diameter) and a polar column (MXT‐1701: 14% cyano‐propyl phenyl, 86% dimethyl polysiloxane, 10 m length, and 180 µm diameter). An FID detector was connected at the end of each column, and the acquired signal was digitized every 0.01 s.

NRF and RF samples of all eight cultivars were subjected to e‐nose analysis. For each sample, 1 g of flour was placed in a 10‐mL glass vial. The headspace extraction was conducted in a septa‐sealed screw cap vial that was equilibrated for 20 min at 60°C. Afterward, the headspace above the sample was injected into the e‐nose at a speed of 500 µL/s with a pressure of 10 kPa, a flow rate of 60 mL/min, and an injection time of 60 s using an automatic headspace sampler (CTC Analytics company, Zürich, Switzerland). The column oven temperature program used for the experiment started at 50°C, held for 2 s, and then ramped at a rate of 3°C/s until it reached 250°C and then held for 5 s. The injection temperature of the injector and detector was set at 240°C and 270°C, respectively.

For calibration of the method, an alkane solution (from *n*‐hexane to *n*‐hexadecane) was used to convert retention time in Kovats indices (KI) to identify possible compound matches using the AroChemBase database (Version 4.6, Alpha MOS Corporation, Toulouse, France). The peak areas indicate the relative concentration of the odor components.

### 2.5 Statistical Analysis

Sensory and instrumental volatile data were analyzed for sample differences using the R statistical computing software (version 4.2.2; R Core Team 2022) to conduct analysis of variance (ANOVA) and least significant difference (LSD) post hoc multiple comparisons tests using the following packages: tidyverse v2.0.0 (Wickham et al. 2019) and agricolae v1.3.5 (de Mendiburu 2021). Principal component analysis (PCA), hierarchical cluster analysis (HCA), and Pearson's correlation were also conducted and visualized using R statistical computing software (version 4.2.2; R Core Team 2022) using the following packages: FactoMineR v2.8 (Lê et al. 2008), ggplot2 v3.5.1 (Wickham 2016), and Hmisc v5.1.2 (Harrell Jr. 2024).

The data from the sensory descriptive analysis were analyzed using ANOVA. The multifactorial ANOVA model included interactions (panelist:sample, panelist:day, and sample:day), with panelists treated as a random effect and sample and day as fixed effects. A pseudo‐mixed model was applied to verify whether sample effects were significant independently of interactions with panelist and day. Sensory attributes with significant panelist or day interaction effects were excluded, and LSD post hoc analysis was performed on the remaining significant sensory attributes to identify differences in attribute ratings between samples. For all statistical tests, an *α* of 0.05 was used to determine statistical significance. Mean intensity ratings from duplicate reps for significantly different sensory attributes were used for PCA analysis to identify relationships among pulse samples based on their sensory attributes, and HCA analysis was conducted to segment samples into subgroups sharing common sensory patterns. Bar plots were generated using Microsoft Excel (Microsoft Corporation, Seattle, WA, USA).

The volatile peak areas from the HS‐SPME–GC–MS analysis represent the average of three replicates (Ponkshe et al. 2025). The identified volatile compounds using HS‐SPME–GC–MS were categorized according to their chemical class as follows: aldehydes, alkanes, alcohols, ketones, terpenoids, sulfurous, nitrogenous, and aromatic compounds, and were analyzed using ANOVA followed by LSD post hoc multiple comparisons tests. The mean‐centered AUC values were analyzed using PCA to examine relationships between volatile profiles and processed pulse samples, as well as HCA to group samples with similar volatile patterns grouped by chemical class.

The peak areas for each discriminant ion in a sample from e‐nose analysis were obtained from the average of triplicates. Partial least squares (PLS) regression was performed using the Alpha MOS software (Version 2023, Toulouse, France) to identify discriminant ions from raw chromatograms by correlating e‐nose volatile profiles with mean DA intensity scores for the following significantly different (*p* < 0.05) aroma attributes: kidney beany, chickpea, pinto beany, great northern beany, boiled rice, toasted bread, tofu, and grainy; and flavor attributes: kidney beany, chickpea, pinto beany, great northern beany, tofu, mushroom, and vegetable flavor (Cevoli et al. 2022; Lozano et al. 2007; Ravi et al. 2019). Alpha MOS software enables the selection of the peaks that show the maximum variability between groups with lowest variability within the same group, where, in this case, groups correspond to aroma and flavor attribute intensities. The process is done by ranking the DI (Discrimination Index) of chromatographic peaks, after which the DI of compounds is scaled from 0 (no statistical difference between groups) to 1 (highest statistical difference between the groups). We applied a threshold of DI >0.95 to select the peaks that are most correlated to the sensory perception. Mean‐centered peak areas of discriminant ions were used for PCA to visualize the relationship between pulse flour and discriminant ions, as well as HCA to segment samples into subgroups sharing common discriminant ion markers.

PCA coordinate distance matrices from the first three dimensions of the following—sensory descriptive analysis mean ratings (DA), mean peak areas of discriminant ions from e‐nose analysis, and means of AUC of volatiles analyzed by HS‐SPME–GC–MS—were used to conduct Pearson's correlation test and depicted in a scatter plot.

## 3 Results and Discussion

### 3.1 DA

Panelists consistently and significantly (ANOVA, *p* < 0.05) differentiated pulse varieties based on appearance, aroma, aroma‐by‐mouth, taste, and aftertaste. The ANOVA results showed that out of 25 sensory descriptors, 20 descriptors were significantly discriminating (*p* < 0.05). The following attributes did not show significance: mushroom aroma, boiled potato aroma, boiled potato flavor, bitter taste, and bitter aftertaste. Mean panel attribute ratings and LSD values for the significantly discriminating (*p* < 0.05) sensory descriptive attributes grouped by modality are reported in Table 2.

**TABLE 2:** Mean ratings on a 0–15 intensity scale for attributes that showed significant differences between samples (ANOVA, *p* < 0.05) from descriptive analysis grouped by modality for non‐roasted porridge (NRP), roasted porridge (RP), and boiled pulse (BP) of eight pulse cultivars: Navy (N), Otebo (O), Cranberry (CR), Chickpea (CHKP), Manteca (MN), Mayacoba (MY), White Kidney (WK), and Great Northern (GN). Means are the average ratings for attributes from nine panelists over two replications. Least significant difference (LSD) and sample effect *p*‐values for each sensory attribute in a column are also reported. For each attribute column, mean values that do not share a letter are significantly different (*p* < 0.05).

Modality: Appearance, taste, and aftertaste					
Sample	Color value	Color saturation	Taste sour	Taste umami	Aftertaste astringent
*p*‐value	6.8 × 10^−81^	4.6 × 10^−67^	8.3 × 10^−5^	5.0 × 10^−14^	2.0 × 10^−7^
LSD value	0.55	0.49	0.99	1.04	1.17
N_NRP	2.25^l^	2.51^jklm^	1.46^bcdefg^	4.61^a^	2.82^bcd^
N_RP	3.01^j^	3.36^fg^	1.97^abcd^	4.69^a^	3.36^abc^
N_BP	2.36^kl^	2.78^hijkl^	0.88^efgh^	1.71^k^	1.41^ef^
CHKP_NRP	4.72^fg^	5.07^b^	1.57^bcdef^	3.94^abcde^	3.39^abc^
CHKP_RP	5.32^de^	5.25^b^	1.44^bcdefg^	4.83^a^	3.97^ab^
CHKP_BP	6.19^c^	7.69^a^	1.47^bcdefg^	3.1^defghij^	2.53^cde^
CR_NRP	7.19^b^	2.33^lm^	1.49^bcdefg^	3.53^bcdef^	4.04^a^
CR_RP	7.51^b^	2.56^ijklm^	1.39^bcdefgh^	3.52^bcdefg^	4.15^a^
CR_BP	8.92^a^	3.15^fgh^	0.58^fgh^	2.91^efghij^	2.33^cde^
GN_NRP	2.57^jkl^	2.48^klm^	1.08^defgh^	4.01^abcd^	2.56^cde^
GN_RP	4.08^hi^	4.03^cde^	1.55^bcdef^	4.41^ab^	2.52^cde^
GN_BP	3.61^i^	4.28^cd^	0.52^gh^	2.41^hijk^	2.29^cdef^
O_NRP	1.64^m^	2.14^m^	1.16^cdefgh^	4.34^abc^	2.07^def^
O_RP	2.79^jkl^	2.99^ghij^	1.24^cdefgh^	3.97^abcd^	3.44^abc^
O_BP	3.59^i^	3.91^de^	0.44^h^	2.27^jk^	1.79^def^
WK_NRP	2.9^jk^	3.01^ghi^	1.86^abcde^	3.34^cdefghi^	2.02^def^
WK_RP	4.74^fg^	4.25^cd^	2.09^abc^	4.58^a^	2.66^cd^
WK_BP	4.82^efg^	5.37^b^	0.71^fgh^	2.31^ijk^	2.01^def^
MN_NRP	3.74^i^	3.56^ef^	1.4^bcdefgh^	4.35^abc^	1.85^def^
MN_RP	5.46^d^	4.52^c^	2.32^ab^	4.91^a^	2.37^cde^
MN_BP	5.07^def^	5.16^b^	0.72^fgh^	2.48^ghijk^	1.87^def^
MY_NRP	3.02^j^	2.86^hijk^	1.17^cdefgh^	2.87^fghij^	1.76^def^
MY_RP	4.3^gh^	3.39^fg^	2.73^a^	3.43^bcdefgh^	2.31^cdef^
MY_BP	3.81^hi^	4.06^cd^	0.52^gh^	2.51^fghijk^	1.16^f^

Modality: Aroma								
Sample	Aroma tofu	Aroma toasted bread	Aroma boiled rice	Aroma cream of wheat	Aroma kidney bean	Aroma chickpea	Aroma pinto bean	Aroma great northern bean
*p*‐value	9.3 × 10^−59^	3.3 × 10^−6^	1.3 × 10^−9^	4.2 × 10^−3^	7.4 × 10^−33^	2.9 × 10^−38^	3.2 × 10^−13^	1.8 × 10^−9^
LSD value	0.95	1.02	1.13	1.26	0.92	1.20	1.12	1.38
N_NRP	4.59^c^	2.94^efghij^	3.53^cd^	3.84^abc^	2.43^hijkl^	2.85^efgh^	2.3^ghi^	4.14^bcdef^
N_RP	2.75^fghij^	3.1^cdefghij^	3.46^cde^	3.07^cdef^	2.63^hijkl^	2.71^efgh^	2.98^defghi^	2.47^hij^
N_BP	1.68^kl^	3.19^cdefghi^	2.37^efg^	2.01^f^	2.63^ghijkl^	3.73^cde^	3.29^cdefgh^	3.69^efghi^
CHKP_NRP	9.63^a^	2.6^ghij^	4.94^ab^	4.41^ab^	1.79^l^	4.41^bc^	2.11^i^	2.16^j^
CHKP_RP	10.24^a^	2.56^hij^	5.43^a^	4.44^a^	2.02^jkl^	5.61^b^	2.69^efghi^	2.57^ghij^
CHKP_BP	3.24^defgh^	4.44^a^	3.34^cdef^	2.66^cdef^	2.47^hijkl^	11.84^a^	1.96^i^	2.38^ij^
CR_NRP	2.81^fghij^	3.13^cdefghij^	2.37^efg^	3.54^abcd^	3.71^cd^	2.85^efgh^	3.02^defghi^	3.68^efghi^
CR_RP	2.46^ghijk^	4.06^abcd^	2.74^defg^	2.83^cdef^	4.68^b^	1.95^gh^	4.49^b^	3.17^fghij^
CR_BP	2.12^ijkl^	3.09^defghij^	2.26^fg^	2.14^ef^	8.25^a^	1.83^h^	6.69^a^	2.75^ghij^
GN_NRP	3.81^cde^	2.17^j^	4.11^bc^	2.78^cdef^	2.18^ijkl^	3.02^defgh^	2.14^i^	3.93^cdefg^
GN_RP	1.95^jkl^	3.06^defghij^	3.08^cdef^	2.95^cdef^	3.31^cdefgh^	2.85^efgh^	3.41^bcdefg^	3.77^defgh^
GN_BP	2.31^hijkl^	3.92^abcde^	3.04^cdef^	2.13^ef^	3.04^defghi^	3.48^cdef^	3.46^bcdef^	6.21^a^
O_NRP	5.63^b^	2.13^j^	3.38^cdef^	3.31^abcde^	2.27^ijkl^	2.51^fgh^	2.23^hi^	4.17^bcdef^
O_RP	2.13^ijkl^	4.12^abc^	2.35^efg^	3.64^abc^	3.64^cde^	2.84^efgh^	4.28^bc^	5.29^abc^
O_BP	2.16^ijkl^	2.93^efghij^	1.85^g^	2.31^def^	2.78^efghijk^	2.39^fgh^	3.05^defghi^	4.58^bcde^
WK_NRP	2.76^fghij^	2.79^fghij^	2.49^defg^	3.16^bcdef^	2.36^ijkl^	3.02^defgh^	2.82^defghi^	4.53^bcdef^
WK_RP	2.99^efghi^	3.59^abcdefg^	2.47^defg^	3.29^abcde^	2.93^defghij^	2.28^fgh^	3.91^bcd^	3.58^efghi^
WK_BP	2.24^ijkl^	3.39^bcdefgh^	2.38^efg^	3.21^abcdef^	4.74^b^	2.89^efgh^	3.49^bcdef^	5.28^abc^
MN_NRP	3.28^defg^	3.76^abcdef^	2.91^defg^	3.26^abcdef^	2.72^fghijk^	3.03^defgh^	2.76^efghi^	4.73^bcde^
MN_RP	3.64^cdef^	4.29^ab^	3.1^cdef^	3.34^abcde^	3.55^cdefg^	3.44^cdef^	3.66^bcde^	4.17^bcdef^
MN_BP	2.01^jkl^	3.7^abcdef^	1.83^g^	2.64^cdef^	3.61^cdef^	3.71^cde^	3.06^defghi^	5.13^abcd^
MY_NRP	3.97^cd^	2.36^ij^	2.76^defg^	3.51^abcd^	2^kl^	3.1^defg^	2.43^fghi^	5.37^ab^
MY_RP	2.43^ghijk^	3.44^abcdefgh^	3.13^cdef^	3.14^cdef^	2.27^ijkl^	2.36^fgh^	2.96^defghi^	3.62^efghi^
MY_BP	1.47^l^	4.07^abcd^	2.28^fg^	2.33^def^	3.97^bc^	4.17^cd^	3.03^defghi^	3.43^efghij^

Modality: Aroma‐by‐mouth							
Sample	Flavor mushroom	Flavor tofu	Flavor vegetable	Flavor kidney bean	Flavor chickpea	Flavor pinto bean	Flavor great northern bean
*p*‐value	7.2 × 10^−10^	2.9 × 10^−49^	5.7 × 10^−11^	5.5 × 10^−26^	9.6 × 10^−56^	2.6 × 10^−10^	8.0 × 10^−9^
LSD value	1.27	0.95	1.10	0.82	0.91	1.02	1.20
N_NRP	4.96^abc^	3.67^bc^	4.16^ab^	2.14^efgh^	3.01^defg^	1.43^gh^	4.3^abc^
N_RP	3.56^defghi^	2.57^defghi^	2.43^efgh^	1.67^gh^	2.66^efghi^	2.47^cdef^	3.22^cdefg^
N_BP	2.94^ghi^	1.65^ij^	1.67^hi^	2.13^efgh^	1.96^ij^	1.65^fgh^	3.41^bcdef^
CHKP_NRP	3.49^defghi^	8.58^a^	1.8^ghi^	1.54^h^	4.66^c^	1.23^h^	2.07^g^
CHKP_RP	2.99^ghi^	9.12^a^	1.31^i^	2.09^efgh^	6.32^b^	2.34^cdefg^	2.27^fg^
CHKP_BP	3.46^efghi^	3.65^bc^	1.83^ghi^	2.2^efgh^	11.45^a^	1.7^fgh^	2.06^g^
CR_NRP	5.43^a^	2.75^cdefgh^	4.14^ab^	3.64^bc^	2.58^efghi^	2.24^defgh^	4.72^a^
CR_RP	5.08^ab^	2.29^efghi^	2.48^efgh^	4.1^b^	1.44^j^	3.28^bc^	2.99^defg^
CR_BP	3.31^fghi^	1.99^ghij^	2.16^fghi^	6.28^a^	2.12^ghij^	4.82^a^	2.69^efg^
GN_NRP	4.13^bcdefg^	3.49^cd^	3.27^bcde^	2.06^efgh^	2.79^efghi^	1.55^fgh^	3.92^abcd^
GN_RP	3.79^cdefgh^	2.32^efghi^	2.58^defgh^	2.49^defg^	2.19^ghij^	2.56^bcdef^	4.11^abcd^
GN_BP	2.36^i^	1.86^hij^	2.02^ghi^	2.48^defg^	2.9^defgh^	2.34^cdefg^	4.41^abc^
O_NRP	4.38^abcdef^	4.45^b^	3.67^abcd^	1.97^efgh^	2.11^ghij^	1.68^fgh^	3.84^abcde^
O_RP	3.39^fghi^	2.94^cdefg^	2.43^efgh^	2.64^def^	2.26^ghij^	2.73^bcde^	4.69^a^
O_BP	2.37^i^	1.87^hij^	1.92^ghi^	2.46^defg^	2.27^ghij^	2.44^cdefg^	4.36^abc^
WK_NRP	5.11^ab^	3.08^cdef^	4.23^ab^	2.47^defg^	2.57^efghi^	2.24^defg^	4.74^a^
WK_RP	4.37^abcdef^	2.76^cdefgh^	2.79^defg^	2.77^de^	2.43^fghi^	2.84^bcd^	4.38^abc^
WK_BP	2.62^hi^	2.54^defghi^	2.5^efgh^	4.11^b^	2.85^defghi^	3.31^bc^	3.99^abcd^
MN_NRP	4.76^abcd^	3.49^cd^	3.95^abc^	1.89^fgh^	2.76^efghi^	1.77^efgh^	4.86^a^
MN_RP	5.21^ab^	3.37^cd^	3.24^bcdef^	3.84^bc^	3.21^def^	3.51^b^	4.16^abcd^
MN_BP	2.84^hi^	2.16^fghij^	2.32^efghi^	3.23^cd^	3.76^cd^	1.81^efgh^	4.63^a^
MY_NRP	4.7^abcde^	3.15^cde^	4.37^a^	2.17^efgh^	2.86^defghi^	1.82^efgh^	4.69^a^
MY_RP	5.38^ab^	2.06^ghij^	3.21^bcdef^	2.73^de^	2.06^hij^	2.39^cdefg^	4.61^ab^
MY_BP	3.25^fghi^	1.24^j^	2.89^cdefg^	3.03^cd^	3.35^de^	1.67^fgh^	4.62^a^

#### 3.1.1 Effect of Processing

**FIGURE 3:**
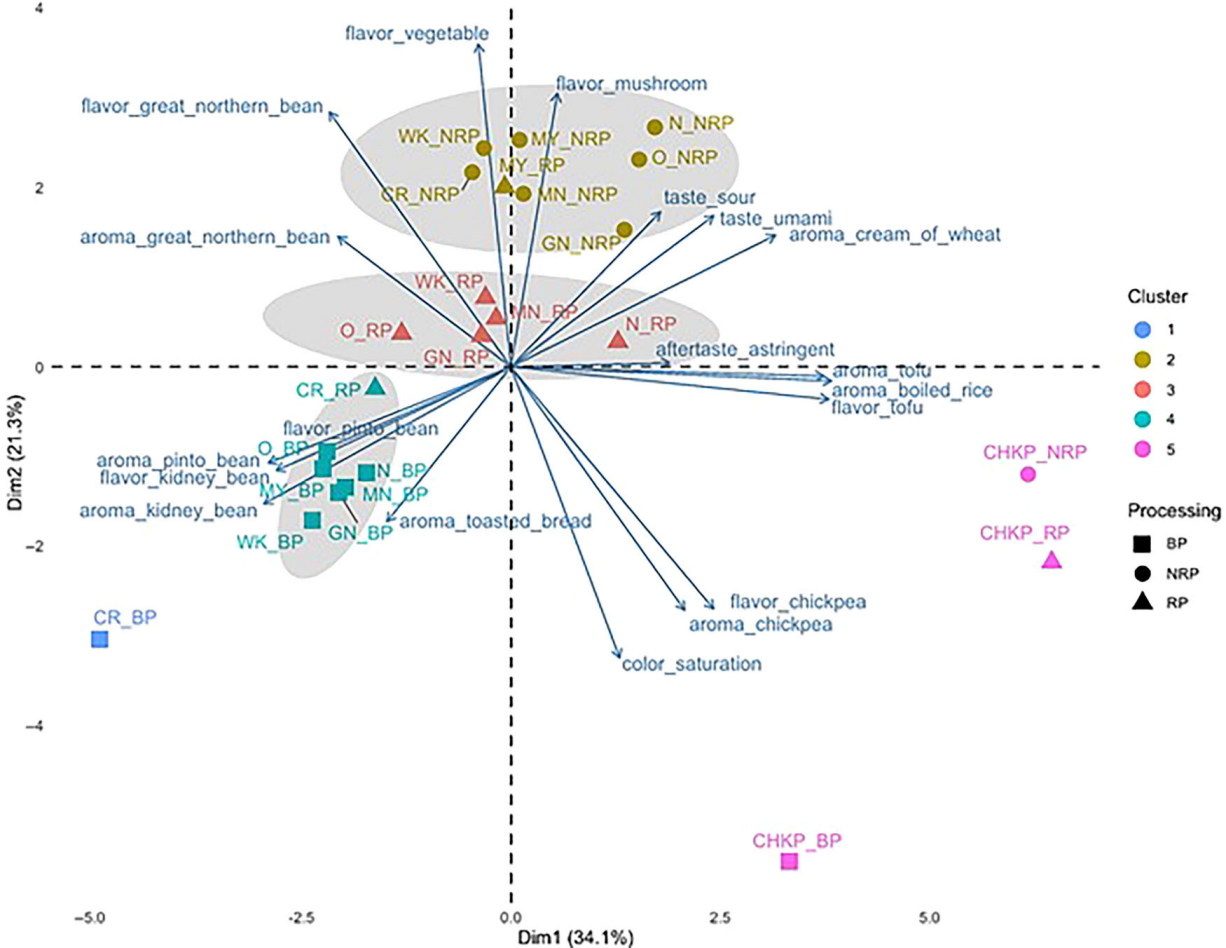
Principal component analysis biplot to visualize the effect of cultivar and processing treatments on significant sensory attributes (*p* < 0.05) in boiled pulse (BP), non‐roasted porridge (NRP), and roasted porridge (RP) represented by squares (■), circles (●), and triangles (▲), respectively, across eight pulse cultivars: Navy (N), Otebo (O), Cranberry (CR), Chickpea (CHKP), Manteca (MN), Mayacoba (MY), White Kidney (WK), and Great Northern (GN). Hierarchical cluster analysis grouped samples into clusters with shared sensory profiles, denoted by color.

HCA highlighted the distinct clustering of pulse samples based on processing treatments. PC1, PC2, and PC3 accounted for 31.4%, 21.3%, and 17% of the variance, respectively, in PCA (Figure 3). BPs were characterized by kidney‐ and pinto‐bean‐like odors and flavors, RP by great‐northern bean‐like odors and flavors, and NRP by vegetative/green and mushroom/earthy/musty flavors. BP samples from white‐colored beans (e.g., Navy, Great Northern, Otebo, White Kidney) and yellow‐colored beans (e.g., Manteca, Mayacoba) (Figure 1) formed cluster 4 in quadrant 3 associated with kidney‐ and pinto‐bean‐like odors and flavors. The panelists characterized the beany notes of great northern bean‐like odor and flavor as sour beany, while the pinto bean‐like odor and flavor were described as sweet and beany. The “beany” odor and flavor ratings for these samples could have stemmed from their closer resemblance to canned bean references provided during the sensory evaluation. These references could have cued visual differences in panelists’ perception, although it also could be that boiling resulted in aroma profiles more similar to canned bean references. Previous literature has characterized boiled beans with beany odor and flavor along with earthy, vegetative notes (Bassett et al. 2021; Koehler et al. 1987; Mkanda et al. 2007).

**FIGURE 4:**
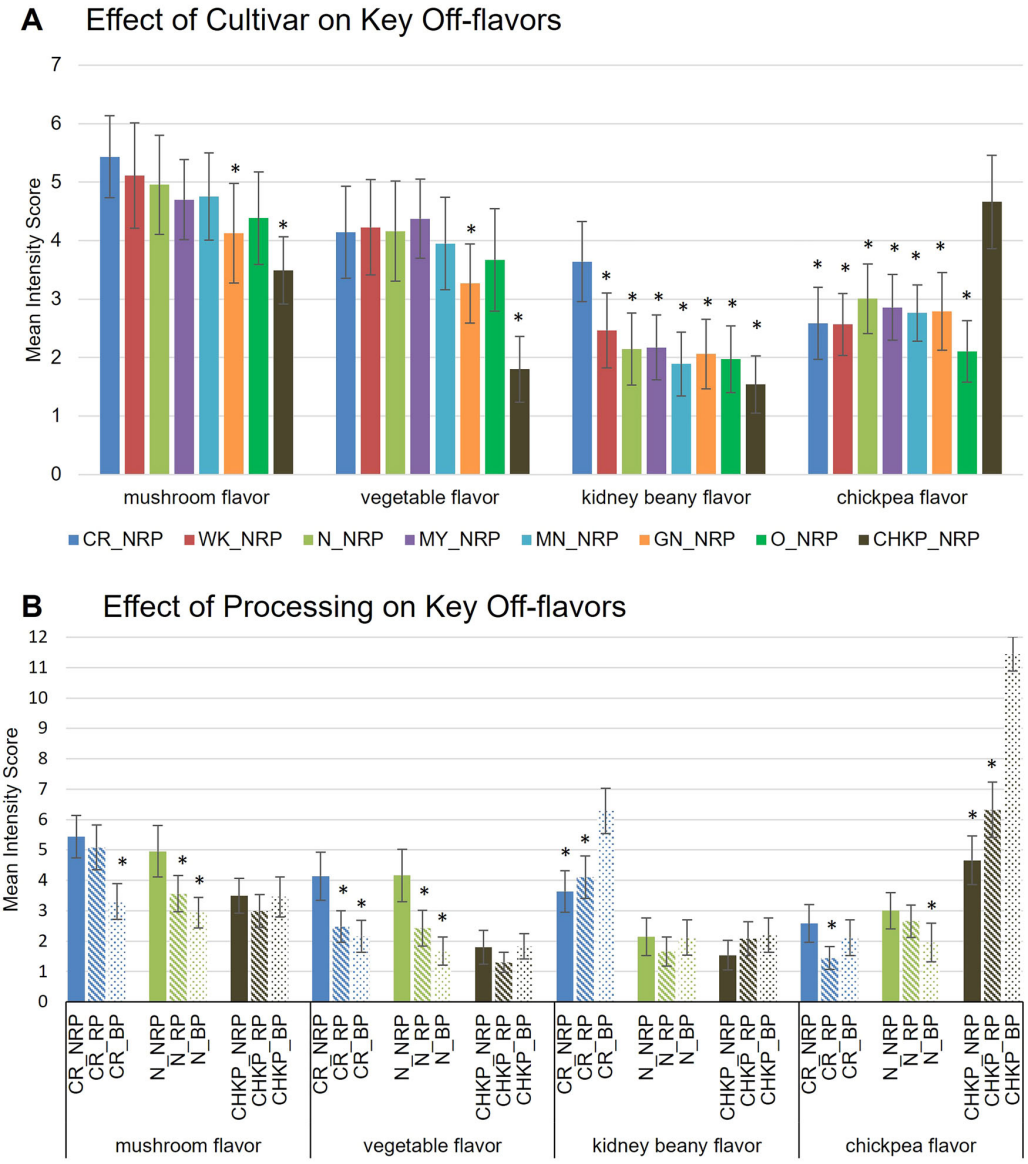
Bar plot displaying the effect of (A) cultivar across eight pulse cultivars—Cranberry (CR), White Kidney (WK), Navy (N), Mayacoba (MY), Manteca (MN), Great Northern (GN), Otebo (O), and Chickpea (CHKP) —and (B) processing treatments in non‐roasted porridge (NRP), roasted porridge (RP), and boiled pulse (BP) on key sensory attributes: earthy/musty/mushroom, green/vegetable, kidney beany, and chickpea flavors. Error bars represent the standard error of DA mean intensity scores. Asterisks denote significantly lower intensity of off‐flavors compared to the sample with strongest intensity—in (A) across cultivars within NRP sample type and in (B) significant reduction in off‐flavors across treatments within a cultivar.

Interestingly, despite NRP and RP samples being visually indistinguishable, PCA revealed a distinct separation between them, confirming that roasting significantly altered the sensory profile of both white and yellow beans. Results revealed that NRP samples of all pulses except Chickpea were strongly associated with “known off‐flavors” including vegetative/green (Troszyńska et al. 2011) and mushroom/earthy/musty flavors (Vara‐Ubol et al. 2004) (Figures 3 and 4A). In contrast, RP samples of white‐colored and Manteca beans were rated higher for “beany odor and flavor” attributes, such as canned great northern bean‐like characteristics (Figure 3; Table 1). This suggests that roasting effectively reduces “known off‐flavors” such as vegetative/green and mushroom/earthy/musty flavors but simultaneously increases some beany attributes (Figure 4B). Previous research supports the benefits of roasting to minimize off‐flavors in pulses before their transformation into food ingredients. For example, Young et al. (2020) demonstrated that roasting peas prior to milling and incorporating the flour into bread reduced beany flavors. Navicha et al. (2018) found that soybeans roasted at 110°C–120°C for extended durations exhibited a marked reduction in beany flavors due to LOX inactivation. Similarly, burgers made with micronized lentil flour exhibited higher consumer acceptability, whereas those made with untreated flour retained a pronounced beany off‐flavor (Der 2010). On the other hand, Ma et al. (2013) found that roasted lentil flour provided the highest flavor scores when used in salad dressings, compared to dressings supplemented with roasted seeds or precooked spray‐dried lentil flour.

Given that our study found both increases and decreases in off‐flavor attribute intensities, these findings highlight the importance of refining pretreatment strategies, such as roasting conditions, including time and temperature, tailored to the specific size and type of pulses, to enhance the flavor profiles of pulse‐based products.

#### 3.1.2 Effect of Cultivar Selection

Among the eight cultivars studied, Chickpea and Cranberry samples exhibited the most distinct sensory profiles, compared to the white and yellow‐colored beans (Figures 1 and 3). For chickpea (BP, RP, NRP), the differences could arise from its classification into a different genus from the rest of the samples—Chickpea (*C. arietinum*) and Common bean (*Ph. vulgaris*), respectively—which could explain their unique sensory characteristics (Figure 3). Panelists rated boiled Cranberry bean samples (cluster 1, quadrant 3) the highest for attributes such as dark color (value), dull appearance (saturation), astringent aftertaste, and strong beany odors and flavors, including kidney‐ and pinto‐bean‐like notes (Figures 3 and 4B). Chickpeas demonstrated distinct sensory characteristics across processing treatments (BP, RP, NRP), consistently clustering separately from other pulses (Figure 4), and stood out for their tofu‐like and canned chickpea–like odors and flavors, forming a cluster 5 in quadrant 4 of the PCA (Figure 4).

Both Chickpea and Cranberry NRP samples received the highest ratings for darkness of appearance and astringent aftertaste among all cultivars during sensory analysis. This astringency may be attributed to their biochemical composition. Nonvolatile compounds, such as isoflavones, saponins, and phenolics, have been associated with bitterness and astringency in soybeans and peas, respectively (Roland et al. 2017). Chickpeas contain phenolic isoflavones, including formononetin and biochanin A, which activate the same bitter receptors as other isoflavones, such as daidzein and genistein, suggesting they may also impart bitterness (Roland et al. 2011). Additionally, phosphatidylcholine, identified in defatted chickpea flour (Sánchez‐Vioque et al. 1998), has been linked to bitterness in soybeans when oxidized (Sessa et al. 1974). This suggests that phosphatidylcholine oxidation in chickpeas may similarly contribute to bitterness. Additionally, dark‐colored pigmented pulses, such as Cranberry beans, exhibited the highest total phenolic levels (19.12 mg/g DW) compared to nonpigmented, lighter‐colored beans such as Navy, Great Northern, Otebo, and White Kidney (Fashakin et al. n.d.) (Figure 1). This highlights the distinct flavor and odor profiles of Cranberry bean and Chickpea compared to white‐colored beans, as in the first row of Figure 1, particularly Navy, Otebo, and Great Northern beans, which exhibited milder sensory attributes (Figures 3 and 4A). This observation aligns with existing literature, which indicates that lighter‐colored beans tend to have milder flavors, making them more versatile for use in food manufacturing. For instance, boiled white‐colored beans were characterized as starchy and sweet with shorter cooking times, whereas dark‐colored beans exhibited stronger vegetative and earthy intensities (Bassett et al. 2021). Studies further support the acceptability of light‐colored beans for use in flour products; for example, a study conducted by Hooper et al. (2023) showed white kidney bean pasta received higher acceptability scores for overall liking and appearance on a 9‐point hedonic scale than darker‐colored Mayacoba and Black bean pasta prototypes. Winged bean seeds with lighter colors were also noted for their mild, nutty flavor, making them generally more acceptable compared to darker, bitter varieties (Ruberte and Martin 1979).

An interesting finding in our study was observed in the Navy bean. Out of all eight cultivars, Navy exhibited significantly reduced “known off‐flavors,” such as mushroom/earthy/musty and vegetative/green/grassy flavors in RP compared to NRP (Chigwedere et al. 2022) (Figure 4B). Although the great northern beany odor was significantly reduced, a small but statistically significant increase in pinto beany flavor was also observed in Navy RP compared to Navy NRP (Figure 4B). These findings highlight the significant impact of cultivar selection on the sensory characteristics of pulse flour. Additionally, promoting the use of light‐colored bean flours, such as Navy, Otebo, and Great Northern, due to their closer resemblance to the color of wheat flour and milder flavor intensity, especially after roasting, could increase their adoption in gluten‐free pulse‐based products as alternatives to the commonly used Chickpea flour (Sadohara et al. 2022) (Figure 4B).

### 3.2 Instrumental Techniques Applied to the Study of Off‐Flavors in Pulses

#### 3.2.1 Volatile Compound Analysis by HS‐SPME–GC–MS

Targeted GC–MS analysis identified 32 volatile compounds, including aldehydes (8), alcohols (6), ketones (4), aromatics (6), terpenoids (1), alkanes (1), nitrogen‐containing compounds (2), and sulfur‐containing compounds (4) (Ponkshe et al. 2025). In total, 12 key volatile compounds were found to be significantly correlated (Pearson's correlation analysis; *p* < 0.05) with odor and flavor intensities assessed by sensory descriptive analysis, highlighting their critical roles in shaping the sensory profiles of pulses through their associations with “known off‐flavors,” such as vegetative/green, mushroom/earthy, and beany attributes. The identified compounds included (*E*)‐2‐hexenal, decanal, benzaldehyde, 1‐hexanol, 1‐octen‐3‐ol, 3‐methyl butanol, styrene, l‐limonene, 2‐pentyl furan, naphthalene, 3,5‐octadien‐2‐one, and 6‐methyl‐5‐hepten‐2‐one.

**FIGURE 5:**
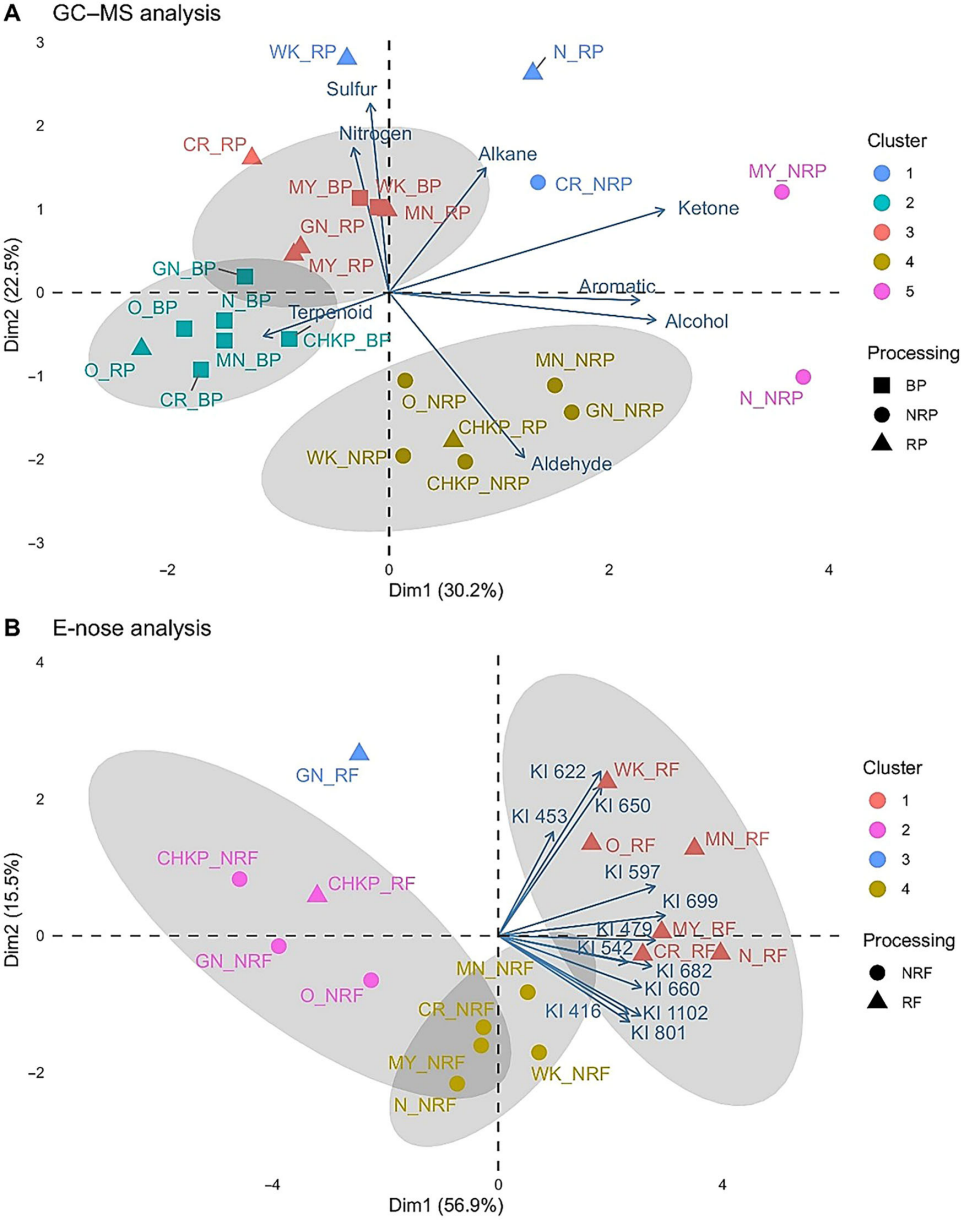
Characterization of pulse flavor via GC–MS and e‐nose. (A) Principal component analysis (PCA) biplot to visualize the relationship between area under the curve of volatiles analyzed by HS‐SPME–GC–MS, grouped by chemical class, and pulse samples in boiled pulse (BP), non‐roasted porridge (NRP), and roasted porridge (RP) represented by squares (■), circles (●), and triangles (▲), respectively, across eight pulse cultivars: Navy (N), Otebo (O), Cranberry (CR), Chickpea (CHKP), Manteca (MN), Mayacoba (MY), White Kidney (WK), and Great Northern (GN). Samples sharing similar volatile profiles are clustered together using hierarchical cluster analysis (HCA) and represented by distinct colored clusters. (B) PCA biplot to visualize the relationship between peak areas of discriminant ions (DI > 0.97), identified through PLS analysis of peak areas from e‐nose analysis and mean panel attribute ratings from sensory descriptive analysis, for non‐roasted flour (NRF) and roasted flour (RF) samples represented by circles (●) and triangles (▲), respectively, for eight pulse cultivars: N, O, CR, CHKP, MN, MY, WK, and GN. HCA grouped samples with similar discriminant ion profiles into distinct colored clusters. Predictive compound identities associated with the discriminant ions are listed in Table S1.

To explore the relationships between volatile profiles of cooked pulse samples (NRP, RP, and BP) across eight cultivars, PCA was conducted. Together, PC1 (30.2%), PC2 (22.5%), and PC3 (15%) explained 67.7% of the variance in the volatile peak areas. Processing treatment drove differences in volatile profiles of the cooked samples (NRP, RP, and BP) such that NRP samples clustered predominantly in quadrants I and IV. In contrast, the thermally processed RP and BP samples are clustered in quadrants II and III, respectively (Figure 5A).

NRP samples from Mayacoba and Cranberry cultivars clustered in quadrant I, forming clusters 1 and 5, respectively, exhibited higher concentrations of alkanes and ketones, while Chickpea, White Kidney, and Great Northern beans in quadrant IV, represented by cluster 4, were associated with aldehydes, alcohols, and aromatics. The NRP samples were mainly characterized by higher concentrations of aldehydes and alcohols (Ponkshe et al. 2025). Alcohols such as 1‐octen‐3‐ol (*R* = 0.67) and 3‐methyl butanol (*R* = 0.41) were significantly correlated with mushroom/earthy/musty flavors, while 1‐hexanol (*R* = 0.61) was significantly correlated with vegetative/green flavor (*p* < 0.05). Similarly, aldehydes including (*E*)‐2‐hexenal (*R* = 0.67) and benzaldehyde (*R* = 0.61) were significantly correlated with vegetative/green flavors, while decanal (*R* = 0.65), benzaldehyde (*R* = 0.64), and (*E*)‐2‐hexenal (*R* = 0.51) were significantly correlated with mushroom/earthy/musty flavors as observed in sensory descriptive data (*p* < 0.05). These findings align with previous studies. For instance, Vara‐Ubol et al. (2004) used sensory descriptive analysis and HS‐SPME–GC–MS to demonstrate that low concentrations (1–10 ppm) of hexanol and 2‐pentyl furan contributed to musty and earthy notes, while hexanal was strongly associated with green/pea pod aromas. Similarly, Xu et al. (2019) identified hexanal (grassy), (*E*,*E*)‐2,4‐nonadienal (rancid), 1‐hexanol (green), 1‐octen‐3‐ol (mushroom), and 2‐pentyl furan (green bean) as key markers of beany flavors in germinated lentil flour using HS‐SPME–GC–MS/olfactometry. In our study, although significant correlations (*p* < 0.05) were observed between individual volatiles and sensory attributes among chemical classes, alcohols uniquely exhibited significant correlations (*p* < 0.05) with both vegetative (*R* = 0.62) and mushroom/musty (*R* = 0.50) flavors, as determined by sensory descriptive analysis. Thus, in less thermally processed NRP samples, alcohol concentration could be the predictive indicator of known off‐flavors in pulses. NRP samples across all pulses except Chickpea demonstrated elevated levels of hexanal, hexanol, 1‐octen‐3‐ol, and 2‐pentyl furan, which corresponded to stronger intensities of vegetative/green and mushroom/earthy off‐flavors in sensory descriptive analysis (Figure 3). Roasting significantly (*p* < 0.05) reduced these volatiles in RP samples, particularly in the Navy cultivar, explaining the lower sensory intensities of vegetative/green and earthy/musty flavors in RP samples compared to NRP samples (Figure 4B). Since the NRP samples were characterized by higher concentrations of aldehydes and alcohols, these results highlight the importance of roasting in reducing known off‐flavors by decreasing the concentrations of key volatiles responsible for vegetative/green and mushroom/earthy flavors. Enzymatic lipid oxidation in legumes involves the formation of fatty acid hydroperoxides by lipoxygenase (LOX), which are subsequently cleaved by hydroperoxide lyase (HPL) into aldehydes. Alcohol dehydrogenase (ADH) can subsequently reduce aldehydes and ketones into their corresponding alcohols (Fischer et al. 2022). Previous studies have shown that heat treatments can effectively disrupt this pathway. For example, Kalua et al. (2007) reported that thermal treatments at 60°C–80°C can partially deactivate the LOX–HPL enzyme system, thereby limiting the formation of volatile aldehydes and alcohols, while Del Rosario et al. (1984) observed significantly reduced levels of alcohols after thermal treatment at 190°C.

In contrast to NRP samples, the thermally treated RP and BP samples showed elevated levels of terpenoids as well as sulfurous and nitrogenous compounds. These nitrogenous and sulfurous compounds can be formed by the Maillard reaction, a nonenzymatic browning process driven by interactions between amino acids and reducing sugars (Mishra et al. 2019; Yu et al. 2021). Specifically, quadrant II predominantly included RP samples from Manteca, Mayacoba, Cranberry, White Kidney, and Great Northern cultivars. These samples exhibited elevated levels of sulfurous compounds such as dimethyl disulfide and methional as well as nitrogenous compounds, which were not detected in NRP samples. Following heat treatment, nitrogenous compounds such as 3‐butyl‐2,5‐dimethyl‐pyrazine and 2,5‐dimethyl‐pyrazine increased significantly (*p* < 0.05) in RF and RP samples of Cranberry, White Kidney, Manteca, and Mayacoba cultivars (Ponkshe et al. 2025). Alkylpyrazines, which contribute a nutty flavor, are primarily formed through Maillard reactions between amino acids and carbohydrates (Shibamoto and Bernhard 1977) or by the pyrolysis of serine and threonine (Baltes and Bochmann 1987) during thermal treatments. Sulfur compounds and pyrazines, despite their low odor thresholds (Landaud et al. 2008; Müller and Rappert 2010), did not show significant correlations with sensory attributes from DA in this study. This limitation may stem from the targeted approach for GC–MS analysis chosen in this study, which may not have encompassed a broader range of sulfurous and nitrogenous compounds that could potentially contribute to the beany odors observed in sensory evaluations. Expanding the scope of targeted compounds in future analyses or leveraging the untargeted profiling could provide a more comprehensive understanding of the volatile markers contributing to beany odors. Quadrant III, on the other hand, consisted mostly of BP samples from Otebo, Navy, Manteca, Chickpea, Cranberry, and Great Northern cultivars. These samples were primarily associated with terpenoid compounds. The presence of monoterpenes such as α‐pinene, β‐pinene, sabinene, 3‐carene, myrcene, limonene, (*Z*)‐β‐ocimene, and (*E*)‐β‐ocimene may originate from endogenous isoprenoid biosynthesis or carotenoid degradation, potentially catalyzed by LOX or hydroperoxides. Terpenoids showed significant positive correlations (*p* < 0.05) with chickpea‐like, kidney bean–like, and pinto bean–like beany odors and flavors from sensory descriptive analysis. Terpenoids have been reported to increase after roasting in flours of navy bean, red kidney bean, and yellow peas (Ma et al. 2016) and after blanching in green peas (Barra et al. 2007; Jakobsen et al. 1998; Oomah et al. 2007). However, these compounds have not been directly linked to producing beany odors and flavors in prior research. In fact, Liu et al. (2023) reported “fragrant” sensory properties of egg white powder linked to high terpene content, and other studies have suggested that compounds such as limonene and linalool could mask unpleasant odors (Ben Salha et al. 2021). This limitation highlights that targeted GC–MS was unable to identify volatiles or chemical classes responsible for beany odors and flavors.

In summary, targeted GC–MS analysis identified 12 key flavor compounds that were significantly correlated (*p* < 0.05) with odor and flavor intensities based on sensory descriptive analysis. The analysis also provided deeper insights into the role of roasting in mitigating aldehydes and alcohols associated with vegetative/green and mushroom/earthy/musty off‐flavors, showcasing the method's capability for precise qualitative and quantitative profiling of volatile compounds.

#### 3.2.2 Volatile Compound Analysis by E‐Nose

Flour samples were analyzed using an e‐nose to determine whether volatile profiles from raw flour could predict the flavor attributes of cooked pulse products, aimed to streamline product development by identifying key markers directly from raw materials. The e‐nose detected 64 major peaks, with 12 peaks identified as discriminant ions through PLS regression analysis of e‐nose data and sensory descriptive assessments. Retention times of these discriminant ions were converted to KI, and potential compound profiles were suggested using the AroChemBase database.

E‐nose distinguished the volatile profiles of NRF and RF samples from eight pulse cultivars, explaining 81% of the variance in discriminant ions across the first three principal components (Figure 5B). NRF samples clustered in quadrants III and IV (Cluster 4), predominantly representing White Kidney, Mayacoba, Manteca, and Cranberry cultivars. These samples were associated with discriminant ions KI‐801, KI‐1102, and KI‐416, potentially linked to aldehydes and alcohols such as hexanal (leafy), nonanal (sweet), methanol (pungent), and butanol (cheese, sweet, oily, medicinal) (AroChemBase) (Table S1). KI‐801 and KI‐1102 were significantly positively correlated (*R* = 0.5, *p* < 0.05) with mushroom/musty flavors identified in sensory descriptive analysis. However, the e‐nose did not identify discriminant ions directly correlated with vegetative/green flavors observed in DA results.

Conversely, e‐nose data aligned well with DA results in identifying markers associated with increased beany odors and flavors after roasting (Figures 2 and 3). RF samples clustered in quadrants I and IV (Cluster 1), primarily including White Kidney and Otebo cultivars. These samples were associated with discriminant ions KI‐622, KI‐650, and KI‐453, tentatively identified as butanals (almond, toasted, malty), furans (beany, sweet, metallic, vegetable), and sulfurous compounds (rotten cabbage, onion) (AroChemBase) (Table S1). Similarly, RF samples from Mayacoba, Manteca, and Cranberry cultivars were linked to ions KI‐479, KI‐597, and KI‐699, potentially linked to butanal (almond, toasted, malty), pentanal (nutty, almond), propanal (nutty, earthy), and sulfurous compounds such as propanethiol (rotten cabbage, onion) and dimethyl sulfide (rotten, sulfurous) (AroChemBase) (Table S1). Among these discriminant ions, KI‐622, KI‐650, KI‐479, KI‐597, and KI‐699 were significantly correlated with toasted odors (*R* = 0.7) and canned kidney bean‐like (*R* = 0.6) and pinto bean–like odors and flavors (*R* = 0.7) from sensory descriptive assessments (*p* < 0.05). These markers, likely linked to butanals (toasted, malty), furans (burnt, sweet), and sulfurous compounds (rotten cabbage, onion), provide insights into volatile compounds contributing to toasted and beany odors and flavors in pulses after roasting. Previous studies have highlighted the role of sulfurous and furan compounds in off‐flavors. Mishra et al. (2019) demonstrated correlation of volatile profile data with sensory descriptive analysis and odor activity values to establish the role of sulfurous compounds, such as methanethiol, diethyl sulfide, dimethyl disulfide, methional, and dimethyl trisulfide, in contributing to “cooked kidney beany” aroma, while dimethyl sulfoxide and dimethyl sulfone were associated with sulfurous odors. Furans are commonly formed through Maillard reactions or the thermal degradation of sugars, amino acids, carotenoids, and polyunsaturated fatty acids (PUFAs) such as linoleic acid (Izzotti and Pulliero 2014; Min et al. 2003). Sharan et al. (2022), Trindler et al. (2022), and Wang et al. (2021) identified 2‐ethyl furan and 2‐pentyl furan as key contributors to earthy, green, and beany notes in peas, faba beans, and soybeans.

#### 3.2.3 Comparing GC–MS and E‐Nose for Rapid Off‐Flavor Profiling in Pulses

**FIGURE 6:**
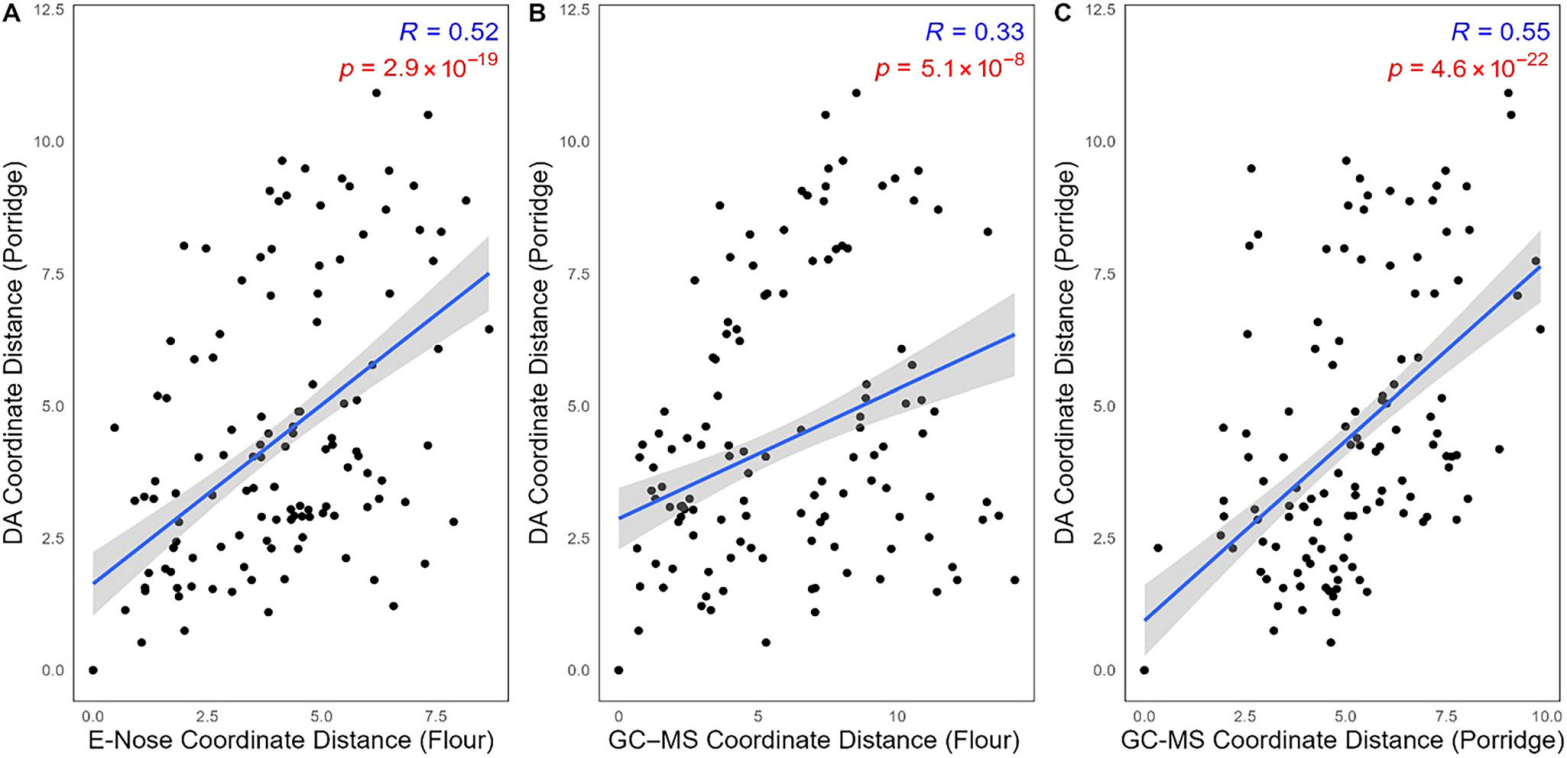
Scatter plots with linear trendlines demonstrating the relationship between principal coordinate analysis (PCA) coordinate distance matrices of sensory descriptive analysis mean ratings (DA) for non‐roasted porridge (NRP) and roasted porridge (RP) and PCA coordinate distance matrices of (A) discriminant ions from e‐nose analysis in non‐roasted flour (NRF) and roasted flour (RF); (B) volatiles analyzed by HS‐SPME–GC–MS in NRF and RF; and (C) volatiles analyzed by HS‐SPME–GC–MS in NRP and RP. *R*‐values indicate the Pearson's correlation coefficient between the two variables depicted in the scatter plot. A *p*‐value of <0.05 indicates statistical significance in predicting sensory attributes.

An objective of this study was to evaluate the effectiveness of GC–MS and e‐nose in predicting off‐flavors in pulses, determining which technique offers better potential for rapid profiling. We evaluated how well profile distances for e‐nose or GC–MS predicted the degree of sensory difference (Figure 6). While DA remains the benchmark for assessing sensory profiles, its reliance on trained panels, extensive sample preparation, and high costs makes it impractical for large‐scale or high‐throughput evaluations (Shurmer and Gardner 1992). Instrumental techniques such as GC–MS and e‐nose address these limitations by offering efficient, reproducible, and time‐saving alternatives for off‐flavor profiling. These methods can identify chemical compounds associated with sensory perception, complementing traditional DA approaches.

A significant correlation (*p* = 4.6 × 10^−22^, *R* = 0.55) was observed between GC–MS volatile profiles and sensory attributes in cooked pulse products (NRP, RP) (Figure 6C). However, its ability to predict sensory characteristics from uncooked pulse flours (NRF, RF) was limited, as evidenced by weaker correlations (*p* = 5.1 × 10^−8^, *R* = 0.33; Figure 6B). In our study, the untargeted e‐nose method enabled the identification of a broader range of volatile compounds, and subsequent PLS analysis aided in the identification of off‐flavor–relevant peaks. As shown in Figure 6A, the e‐nose demonstrated a significant correlation (*p* = 2.9 × 10^−19^, *R* = 0.52) between discriminant ions in uncooked pulse flours (NRF, RF) and sensory data for cooked products (NRP, RP) (Figure 6A). The discriminatory ions KI‐622, KI‐650, KI‐453, KI‐479, KI‐597, and KI‐699, identified by e‐nose, could serve as a digital fingerprint for beany odors and flavors. This ability to predict beany notes directly from raw pulse flour without cooking makes the e‐nose a valuable tool for rapid screening. This can allow breeders and product developers to rapidly identify cultivars or formulations with reduced off‐flavors, eliminating the need for extensive sample preparation and cooking. Additionally, an e‐nose has been reported to facilitate the optimization of processing parameters, such as roasting time and temperature, to minimize the formation of undesirable volatile compounds. Previously, Cai et al. (2021) investigated the effects of various roasting times and temperature levels on the physicochemical, sensory, and volatile profiles of soybeans using both e‐nose and HS‐SPME–GC–MS techniques. Similarly, Asikin et al. (2018) compared the ripening stages of dogfruit (*Pithecellobium jiringa*) and stink bean (*Parkia speciosa*) using HS‐SPME–GC–MS and an MS‐based e‐nose. The results from these studies concluded that HS‐SPME–GC–MS identified specific compounds, providing detailed chemical profiles and insights into the aroma changes. In contrast, the e‐nose analysis generated discriminant ions that enabled rapid differentiation of ripening stages and pretreatment conditions through multivariate analysis (Asikin et al. 2018; Cai et al. 2021).

E‐nose's ability to analyze overall aroma profiles highlights its strength in rapid screening and quality control, particularly for industrial applications. Key advantages of e‐nose include high sensitivity, rapid analysis times, and ease of use, making it a practical tool for settings far removed from specialized chemical laboratories (Dymerski et al. 2011; Otles 2016; Van Ruth 2001). Despite these advantages, GC–MS remains an essential tool for quantifying specific volatile changes and understanding the effects of processing on pulse volatiles, such as those induced by roasting. These approaches offer a robust strategy for optimizing product development and quality control in pulse‐based foods, enabling both rapid screening and detailed characterization of volatile profiles.

## 4 Conclusion

This study investigated the sensory characteristics and volatile profiles of eight pulse cultivars to address challenges associated with off‐flavors and processing in pulse‐based products, while also evaluating the potential of instrumental approaches to predict flavor development. Sensory and volatile differences across cultivars and processing methods were observed using DA, GC–MS, and e‐nose. Sensory analysis revealed that cultivars were differentiated primarily based on appearance and seed coat characteristics. The sensory and volatile profiles following processing pretreatments, such as roasting and boiling, demonstrated shifts in the flavor profiles of treated pulse samples, with some flavors reducing and others intensifying as a result of heat treatment.

Among the cultivars studied, navy bean flour exhibited milder flavor intensities because roasting, in particular, significantly shifted its sensory profile by reducing alcohols and aldehydes responsible for undesirable vegetative and mushroom‐like flavors to beany and toasted‐bread‐like notes linked to furans, sulfurs, and pyrazines. In addition to reducing known off‐flavors, roasting presents a more scalable, energy‐efficient, and nutrient‐preserving processing strategy compared to boiling, making it the recommended treatment for improving the sensory quality of pulse flours. However, this study did not examine year‐to‐year variations, which may influence volatile composition and, consequently, flavor profiles. Future research can investigate the impact of environmental factors on flavor variability in pulse cultivars.

In evaluating instrumental predictors of flavor, e‐nose successfully captured key beany flavor markers, aligning with DA findings better than targeted GC–MS, demonstrating its potential as a predictive tool for rapid flavor profiling in pulses. However, the untargeted approach of e‐nose may have cast a wider net, detecting broader classes of discriminant ions potentially arising from furans or sulfurs that were underrepresented during targeted GC–MS analysis. Additionally, differences in column polarity between the two instruments could have influenced volatile separation and detection. Another limitation is that peak area was used to represent volatile content rather than a metric such as the relative odor activity value (ROAV), which accounts for differences in odor detection thresholds. While the use of ROAV analysis is common (Aisala et al. 2019; Xiao et al. 2024) and valuable for identifying compounds present at detectable levels, it is an imperfect predictor of sensory intensity. Finally, since model products (roasted and non‐roasted porridges) were not analyzed using e‐nose, it is difficult to conclusively determine its superiority over GC–MS in predicting sensory characteristics of finished products.

The findings provide a foundational understanding of how cultivar selection, heat processing, and volatile composition influence the sensory quality of pulses. Future research should explore the impact of different milling techniques on flavor profiles. Investigating the effects of alternative pretreatment methods, such as infrared radiation or optimized roasting conditions, by leveraging an e‐nose as a rapid screening tool, can help identify processing conditions that enhance sensory quality. Identifying cultivars tailored for specific product applications could significantly improve consumer acceptance. Furthermore, consumer testing is needed to evaluate whether the sensory profile changes resulting from processing are perceived positively or negatively, particularly in the context of targeted food applications such as snacks, pastas, or baked goods.

This study highlights the complementary roles of GC–MS and e‐nose techniques in refining pulse flour flavor profiles. By providing actionable insights into optimizing processing parameters and cultivar selection, these findings contribute to the integration of pulse flours into diverse food products, increasing pulse consumption while addressing global food security challenges.

## Author Contributions

**Kaveri Ponskhe**: methodology, investigation, validation, formal analysis, data curation, visualization, writing – original draft. **Aubrey Dubois**: project administration, formal analysis, visualization, writing – review and editing, methodology. **Lili Towa**: methodology, investigation, formal analysis, writing – review and editing, writing – original draft. **Sharon Hooper**: investigation, methodology, writing – review and editing, supervision. **Karen Cichy**: conceptualization, funding acquisition, methodology, writing – review and editing, resources. **Emily J. Mayhew**: conceptualization, funding acquisition, methodology, writing – review and editing, visualization, formal analysis, supervision.

## Conflicts of Interest

E‐nose data were collected using the AlphaMos system, and L.T. was an employee at AlphaMos during the period of data collection and analysis. The authors declare no other conflicts of interest.

## Supporting information



Supplementary Table: jfds70610‐sup‐0001‐TableS1.docx

## Data Availability

Raw data will be made available upon request. After publication, raw data and data analysis scripts will be shared via a public GitHub repository.
