# Supplementary material for: Evaluating the Impact of Cultivar and Processing on Pulse Off‐Flavor Through Descriptive Analysis, GC–MS, and E‐Nose
Source: J Food Sci. 2025 Oct 24;90(10):e70610. doi: 10.1111/1750-3841.70610 (PMC12552770; doi:10.1111/1750-3841.70610)
Supplement: Supplementary file 1 — Supplementary Table: jfds70610‐sup‐0001‐TableS1.docx [file JFDS-90-0-s001.docx]

**Table S1:** Discriminant ions (DI) profiled in pulse flour using e-nose. The results shown are the average of triplicate measurements of peak areas from discriminant ions for non-roasted flour (NRF) and roasted flour (RF) of eight pulse cultivars: Navy (N), Otebo (O), Cranberry (CR), Chickpea (CHKP), Manteca (MN), Mayacoba (MY), White Kidney (WK), Great Northern (GN). Potential compound profiles and odors associated with the respective DI were identified using the AroChemBase database (Version 4.6, Toulouse, France).

| **DI** | **Area of discriminant peaks** | | | | | | | | | | | | | | | | | **Profiled compounds identified using AroChemBase V7 database** | |
| --- | --- | --- | --- | --- | --- | --- | --- | --- | --- | --- | --- | --- | --- | --- | --- | --- | --- | --- | --- |
| **KI** | **N_**  **NRF** | **N_**  **RF** | **CHKP_**  **NRF** | **CHKP_**  **RF** | **CR_**  **NRF** | **CR_**  **RF** | **GN_**  **NRF** | **GN_**  **RF** | **O_**  **NRF** | **O_**  **RF** | **WK_**  **NRF** | **WK_**  **RF** | **MN_**  **NRF** | **MN_**  **RF** | **MY_**  **NRF** | **MY_**  **RF** | **Odor description** | |  |
| **453** | 12278 | 19326 | 9550 | 19425 | 5394 | 15683 | 9926 | 13575 | 13022 | 14497 | 16353 | 67702 | 10487 | 16886 | 10666 | 16951 | acetaldehyde (aldehydic, fruity…); methanethiol (sulfurous…) | |  |
| **478** | 112750 | 133015 | 42872 | 51490 | 90084 | 121512 | 83894 | 86030 | 82998 | 137414 | 107212 | 125410 | 128736 | 127389 | 95010 | 115969 | propanal (nutty, earthy...); dimethyl sulfide; | |  |
| **542** | 11494 | 14390 | 8340 | 13269 | 9061 | 13344 | 8100 | 9031 | 9885 | 11855 | 15574 | 12358 | 12344 | 14190 | 11237 | 12390 | 2-methylpropanal ( fruity, malty, toasted…); 1-propanol (alcoholic, ethereal…) | |  |
| **596** | 8186 | 17927 | 7681 | 7992 | 13450 | 16493 | 10171 | 10600 | 7718 | 16889 | 10829 | 13604 | 12225 | 19642 | 11209 | 19731 | butanal (malty, malty, pungent...); 1-propanethiol (cabbage, onion...); 2-Butanone (chocolate, butter, fruity…) | |  |
| **609** | 6090 | 0 | 0 | 0 | 0 | 0 | 0 | 4674 | 0 | 1274 | 0 | 4237 | 0 | 0 | 0 | 0 | ethyl acetate(apple, fruity...); butan-2-one (cheese, sharp…) | |  |
| **621** | 0 | 7902 | 4692 | 5858 | 2958 | 8063 | 1576 | 7929 | 3669 | 10107 | 4325 | 9556 | 2946 | 11058 | 2686 | 7729 | 2-methylfuran (chocolate, burnt, sweet...); but-2-enal (floral, pungent...) | |  |
| **650** | 0 | 8361 | 0 | 0 | 1470 | 6131 | 0 | 11865 | 0 | 7697 | 0 | 6646 | 2582 | 9403 | 0 | 6541 | 3-methylbutanal (almond, toasted, malty...); | |  |
| **659** | 8126 | 11405 | 5688 | 6879 | 6841 | 10614 | 3276 | 0 | 6804 | 8961 | 8996 | 9879 | 6936 | 10068 | 7737 | 10312 | 2-methylbutanal (almond, toasted, malty...); 1- butanol (cheese,strong, sweet, oily, medicinal… ) | |  |
| **681** | 6584 | 9877 | 1431 | 1311 | 8900 | 8537 | 3044 | 3491 | 4117 | 7121 | 6668 | 10766 | 12142 | 11033 | 8898 | 9767 | pent-1-en-3-ol (burnt...); 3-methyl-1-butanol | |  |
| **698** | 4921 | 9183 | 3894 | 3255 | 6520 | 7102 | 1908 | 3973 | 4260 | 7600 | 5094 | 7244 | 7266 | 9721 | 5627 | 8913 | pentanal (nutty, almond...) | |  |
| **732** | 2687 | 7575 | 4905 | 6393 | 3264 | 7510 | 2613 | 7454 | 3959 | 7499 | 4291 | 8379 | 2469 | 9555 | 2890 | 8316 | propanoic acid (soy, rancid, pungent…); pyrazine (roasted, nutty, pungent...); 2- ethyl furan (malty, sweet, burnt…) | |  |
| **800** | 17898 | 25758 | 10419 | 12102 | 24796 | 16261 | 5330 | 8713 | 14535 | 14709 | 17381 | 18531 | 21000 | 21332 | 23965 | 22591 | hexanal (leafy, sharp...); 2-methylpropanoic acid | |  |
| **855** | 853 | 1874 | 703 | 947 | 941 | 984 | 526 | 308 | 653 | 598 | 765 | 1475 | 615 | 1201 | 1245 | 1589 | acetate 2-pentanol (beany, fruity, vegetable...); | |  |
| **913** | 6549 | 12317 | 4029 | 7064 | 6797 | 6517 | 569 | 1381 | 4367 | 5186 | 4565 | 10632 | 4693 | 8567 | 10094 | 10994 | 2,5-dimethylpyrazine (nutty...); ethyl pyrazine; methylthio-propanol (vegetable, cooked potato, ) | |  |
| **953** | 895 | 833 | 1050 | 1095 | 759 | 974 | 1193 | 1021 | 1200 | 858 | 789 | 967 | 1156 | 1157 | 989 | 1095 | benzaldehyde (almond...); a-pinene | |  |
| **991** | 13030 | 16128 | 5744 | 8203 | 9721 | 16563 | 6522 | 6809 | 11905 | 12550 | 11956 | 11300 | 9907 | 14110 | 11226 | 16307 | 2-pentylfuran (beany, sweet, metallic, vegetable…); 1-Octen-3-ol (earthy, fatty, grassy…); | |  |
| **1046** | 4443 | 4491 | 1475 | 2193 | 4661 | 4749 | 2628 | 2796 | 4713 | 4301 | 4550 | 4370 | 4295 | 4384 | 4811 | 4616 | acetophenone (almond, cheese, musty, sweet...); 2-octenal (walnut, earthy...); limonene | |  |
| **1102** | 8750 | 12455 | 1919 | 3636 | 7950 | 11614 | 5728 | 6391 | 6654 | 8923 | 11102 | 5369 | 6654 | 9382 | 8866 | 10460 | 2-isopropyl-3-methoxypyrazine (pea, beany...); tetramethylpyrazine (nutty, burnt...); n-nonanal (sweet...) | |  |
